# Chasing Particularities of Guanine- and Cytosine-Rich DNA Strands

**DOI:** 10.3390/molecules25030434

**Published:** 2020-01-21

**Authors:** Marko Trajkovski, Janez Plavec

**Affiliations:** 1 Slovenian NMR Centre, National Institute of Chemistry, Hajdrihova 19, SI-1000 Ljubljana, Slovenia; janez.plavec@ki.si; 2 EN-FIST Centre of Excellence, Trg OF 13, SI-1000 Ljubljana, Slovenia; 3 Faculty of Chemistry and Chemical Technology, University of Ljubljana, Večna pot 113, p. p. 537, SI-1000 Ljubljana, Slovenia

**Keywords:** G-quadruplex, DNA, double-strand, hybridization, abasic residue, structure, NMR

## Abstract

By substitution of natural nucleotides by their abasic analogs (i.e., 1′,2′-dideoxyribose phosphate residue) at critically chosen positions within 27-bp DNA constructs originating from the first intron of *N-myc* gene, we hindered hybridization within the guanine- and cytosine-rich central region and followed formation of non-canonical structures. The impeded hybridization between the complementary strands leads to time-dependent structural transformations of guanine-rich strand that are herein characterized with the use of solution-state NMR, CD spectroscopy, and native polyacrylamide gel electrophoresis. Moreover, the DNA structural changes involve transformation of intra- into inter-molecular G-quadruplex structures that are thermodynamically favored. Intriguingly, the transition occurs in the presence of complementary cytosine-rich strands highlighting the inability of Watson–Crick base-pairing to preclude the transformation between G-quadruplex structures that occurs via intertwining mechanism and corroborates a role of G-quadruplex structures in DNA recombination processes.

## 1. Introduction

Non-canonical DNA structural motifs have been established as key biological elements [1,2,3]. Particular attention goes to the two groups of four-stranded helical structures termed G-quadruplexes and i-motifs adopted by guanine (G)- and cytosine (C)-rich DNA, respectively [4,5]. G-quadruplexes exhibit stacked G-quartets, which are assemblies comprising four guanine residues in a co-planar arrangements held together by Hoogsteen hydrogen bonds. The formation of G-quadruplexes depends on the presence of (monovalent) cations, which compensate the electrostatic repulsions arising from the closely spaced carbonyl oxygen atoms of stacked G-quartets. Cations, by means of their nature and concentration, also affect the equilibria between different G-quadruplex folds [6]. It is well established that availability of biologically relevant cations, such as K^+^ and Na^+^ ions greatly impacts structural characteristics of G-rich DNA [7,8,9,10,11]. In comparison, the boundary of i-motif formation is determined by solution pH value, which governs formation of the essential structural elements, i.e. intercalated hemiprotonated C^+^·C base pairs. Notably, in addition to i-motif, C^+^·C base-pairing is common also to particular hairpin structures [12,13]. It is the delicate susceptibility of G- and C-rich DNA structural features to micro-environmental conditions that greatly contributes to consideration of these structures as biorecognition elements. Additionally, specific proteins were shown to bind and modulate structural features of G- and C-rich DNA [14,15,16,17,18,19], many of them exhibiting high G-quadruplex and i-motif forming potential. Furthermore, meaningful enrichment of G- and C-rich tracts exists within important genome loci [20,21,22,23,24,25]. The structural insights into the intramolecular motifs of G- and C-rich DNA as oppose to the canonical double-stranded helix have been provided for model systems [26] as well as biologically important regions [27,28,29,30,31,32,33,34,35]. Moreover, previously demonstrated transitions from double-stranded helix to non-canonical structure mediated by G-quadruplex-interacting ligands highlighted particular G-rich segments, e.g., telomeric DNA and promoter region of *c-myc* oncogene as relevant targets for novel anticancer therapeutic approaches [36,37,38,39,40]. This phenomenon led to the quest for ligands that specifically bind to G-quadruplexes and i-motifs [41,42]. However, in most cases, the ligand-design strategy is based on the system comprising the target DNA in the absence of the complementary strand. One of the notable contributions from previous studies is the demonstrated deviation between non-canonical structures in context of single-stranded versus double-stranded DNA [43]. The focus on the insulin-linked polymorphic region revealed that formation of four-stranded G- and C-rich structures in complementary strands are mutually exclusive [44]. The need for more rigorous understanding of single-strand characteristics within a double-stranded DNA was furthermore addressed by studies on constructs comprising joint modules of G-quadruplex, duplex and i-motif forming DNA [45,46,47,48]. Breakthroughs have been achieved by providing high-resolution insights into quadruplex-duplex complexes [49,50], the disclosure of mutual dependence of transcription events and G-quadruplex formation [51] as well as by scrutiny of DNA duplex destabilization occurring in the close proximity to G-quadruplex or i-motif forming region [52]. Nevertheless, the complexity of G- and C-rich DNA intramolecular assembly in the context of double-stranded DNA remains to be much more comprehensively addressed. It is important to explore not only G-quadruplex and i-motif structures ‘framed’ within canonical double-stranded DNA, but also the particular assemblies that can form in parallel and/or in alternative manner to hybridization between complementary G- and C-rich DNA. 

**Figure 1:**
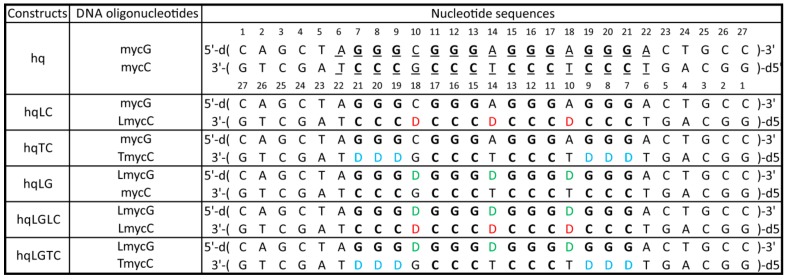
Constructs of DNA oligonucleotides with corresponding primary sequences. The underlined residues within the central part of mycG and mycC mark the segment originating from the first intron of *N-myc* gene. Guanine and cytosine residues within GGG and CCC tracts are in bold. The abasic residues (1′,2′-dideoxyribose) within the sequences of LmycG, LmycC and TmycC oligonucleotides are designated with ‘D’ and colored in green, red and blue when positioned between GGG, CCC and instead of cytosine residue within CCC tracts, respectively.

This study focuses on structural specifics of G- and C-rich DNA oligonucleotides in equimolar mixture of complementary strands, while considering that they can fold into G-quadruplex or i-motif structures when studied alone. DNA constructs of same lengths were designed with the aim to hamper hybridization via Watson–Crick base-pairing and in turn expose single-stranded features of individual oligonucleotide (Figure 1). The central 17-nt segment of the studied constructs originates from the first intron of *N-myc* gene that includes GGG and CCC tracts in the sense and antisense strands, respectively. Moreover, the central part of the G-rich oligonucleotides studied herein corresponds to the core of 19-nt DNA, which in aqueous solution containing K^+^ ions adopts unimolecular G-quadruplex [53]. In this study, the central 17-bp G/C-rich segment (residues 6–22, Figure 1) was extended at both 5′- and 3′-ends by five base-pairs designed to guide hybridization of complementary oligonucleotides. The reference construct ‘hq’ corresponds to equimolar mixture of mycG and mycC oligonucleotides, which are fully complementary with respect to the canonical Watson–Crick base-pairing. To study the roles of nucleobases in the regions between GGG and CCC tracts, we designed oligonucleotides LmycG and LmycC carrying abasic residues (i.e., 1′,2′-dideoxyribose phosphate residue) at positions in the sequences that could be expected to form loop residues, at least in structures of individual strands. Furthermore, to derive a C-rich strand with diminished potential of forming i-motif structures, we synthesized oligonucleotide TmycC distinguished from mycC by cytosine-to-abasic residue substitutions in the first and the fourth CCC tracts. mycG and mycC as well as abasic-residues-comprising LmycG, LmycC and TmycC were used to design six constructs in which G- and C-rich strands were combined in equimolar mixtures (Figure 1). Effects of variations within the C-rich and without modulation of the G-rich strands are addressed in the constructs designated hqLC and hqTC. Moreover, hqLC comprises mycG and LmycC, which exhibits abasic residues between CCC tracts envisaged to impede hybridization between the strands, while minimally perturbing i-motif formation of C-rich strand. On the other hand, hqTC comprises mycG and TmycC, which in comparison to mycC exhibits abasic instead of cytosine residues in the first and the terminal CCC tracts. Expectedly, formation of double-stranded helix in hqTC was to be diminished due to the six abasic sites in the C-rich strand, which were expected also to profoundly reduce i-motif formation ability. The constructs hqLG, hqLGLC, and hqLGTC all comprise LmycG as the G-rich strand, which in comparison to mycG, exhibits abasic instead of natural residues connecting GGG tracts. Notably, the abasic modifications in LmycG were expected not to impart G-quadruplex forming potential, as inferred from the flexibility of the loops in previously reported structure of N-myc [53]. Thus, LmycG was employed in design of constructs in which complementarity between G- and C-rich DNA oligonucleotides decreases, without declining the potential for G-quadruplex formation. Solution-state NMR, CD spectroscopy and native polyacrylamide gel electrophoresis (PAGE) were used to investigate characteristics of individual oligonucleotides and thereupon interpret G- and C-rich strand features occurring in parallel or alternatively to double-stranded helix formation.

## 2. Results

### 2.1. Abasic Loop Residues Aggravate i-Motif, but Promote G-Quadruplex Formation

**Figure 2:**
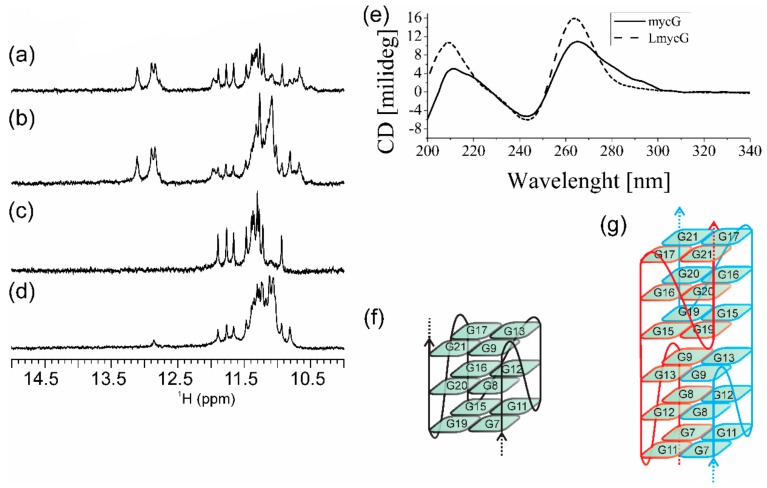
Imino region of ^1^H-NMR spectra of (**a**,**b**) mycG and (**c**,**d**) LmycG. Panels (**a**) and (**c**) correspond to samples recorded immediately after annealing, while panels (**b**) and (**d**) correspond to the spectra recorded 1 day after annealing. The ^1^H-NMR spectra were recorded on an 800 MHz NMR spectrometer at 25 °C and 0.2 mM oligonucleotide concentration per strand. (**e**) CD spectra of mycG (solid line) and LmycG (dashed line) recorded at 20 μM oligonucleotide concentration per strand. The NMR and CD spectra were recorded at 20 mM potassium-phosphate buffer (pH 6.5) and 100 mM KCl. Schematic presentation of kinetically and thermodynamically favored G-quadruplexes adopted by mycG and LmycG are shown in (**f**) and (**g**), respectively.

In the absence of K^+^ ions, mycG alone adopts secondary structures comprising Watson–Crick and non-canonical hydrogen-bonding as inferred from the imino ^1^H-NMR signals at δ12.76, 12.89 and 13.11 ppm as well as at δ10.74, 10.86 and 11.83 ppm (Supplementary Materials Figure S1). The number of imino ^1^H-NMR signals together with their (wide) chemical shift range suggests equilibrium amongst different structures. Upon addition of K^+^ ions into aqueous solution of mycG, new ^1^H-NMR signals appear in the range from δ10.50 to 12.00 ppm, indicating that mycG adopts G-quadruplex structures (Figure 2a). The co-existence of imino ^1^H-NMR signals corresponding to base-pairing in Hoogsteen and Watson–Crick geometries is consistent with equilibrium between different structures, which as indicated by PAGE (vide infra) exhibit different number of monomeric units. Signals in ^1^H-NMR spectra of mycG changed with time indicating that the kinetically predominant G-quadruplex became a minor species one day after addition of K^+^ ions (Figure 2b). Moreover, analysis of the distinct sets of ^1^H-NMR signals corresponding to Hoogsteen hydrogen-bonded imino protons suggests the formation of two different G-quadruplex structures distinguished by different folding kinetics.

LmycG exhibits four GGG tracts linked with abasic residues at positions 10, 14 and 18 (Figure 1). In the presence of K^+^ ions LmycG adopts G-quadruplex structure as demonstrated by twelve imino ^1^H-NMR signals in the range from δ10.90 to 11.90 ppm (Figure 2c). The number and the characteristic ^1^H-NMR chemical shift range of imino signals are consistent with formation of intramolecular G-quadruplex exhibiting three G-quartets. There is almost a perfect match in the imino ^1^H-NMR chemical shifts of the predominant G-quadruplex species in the freshly prepared samples of mycG and LmycG, suggesting that the kinetically favorable structures of both oligonucleotides exhibit the same folding topology. As assessed by ^1^H-NMR monitoring, the structural changes of LmycG over the 24 hour period comprise transformation of the kinetically favored to a distinctive thermodynamically favored and predominant G-quadruplex species (Figure 2d). According to the resemblance of the imino ^1^H-NMR chemical shift regions corresponding to kinetically and thermodynamically predominant species of LmycG and mycG, both oligonucleotides undergo the same structural transitions. The similarity in structural features of mycG and LmycG are corroborated by their CD profiles (Figure 2e). Moreover the maxima and minima at around 265 and 245 nm in the CD spectra of mycG and LmycG are consistent with formation of G-quadruplex with all strands in a parallel orientation. Notably, the shoulder in CD spectrum of mycG above 280 nm is consistent with the presence of a minor species, which may correspond to non-G-quadruplex structures observed by NMR.

**Figure 3:**
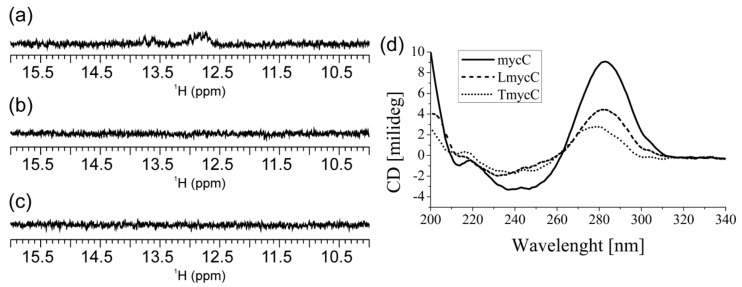
Imino region of ^1^H-NMR spectra of (**a**) mycC, (**b**) LmycC and (**c**) TmycC recorded on 800 MHz NMR spectrometer at 25 °C and 0.2 mM oligonucleotide concentration per strand. (**d**) CD spectra of mycC (solid line), LmycC (dashed line) and TmycC (dotted line) at 20 μM oligonucleotide concentration per strand. The NMR and CD spectra were recorded at 20 mM potassium-phosphate buffer (pH 6.5) and 100 mM KCl.

mycC alone (i.e., in the absence of complementary G-rich oligonucleotide) at pH 6.5 exhibits imino ^1^H-NMR signals of very weak intensity in the region between δ12.60 and 13.80 ppm indicative of secondary structures comprising Watson–Crick base pairs (Figure 3a). No spectral changes are observed for mycC upon 24 h ^1^H-NMR monitoring from annealing. Notably, ^1^H-NMR spectrum of mycC at pH 6.5 lacks the signals at around δ 15.50 ppm that is a fingerprint for i-motif structures stabilized by C^+^·C base pairs. However, the characteristic imino ^1^H-NMR signals are evident in spectrum of mycC at pH 5.0 (Figure S2). LmycC differs from mycC by comprising abasic link between the CCC tracts (Figure 1). On the other hand, TmycC differs from mycC by abasic linkages instead of the first and the fourth CCC tracts (Figure 1). ^1^H-NMR spectra of LmycC and TmycC at pH 6.5 lack signals for C^+^·C base-pairing (Figure 3). However, these results do not rule out transient formation of i-motif structures, whereby broadening of imino ^1^H-NMR signals at pH 6.5 might occur due to the intermediate exchange on the chemical shift time-scale of the NMR spectrometer. In fact, CD spectra of mycC and of the modified LmycC and TmycC exhibit maxima at around 283 nm (Figure 3d) in accordance with i-motif structure formation [54]. CD spectrum of TmycC exhibits maximum with a slight shift from 283 to 278 nm in comparison to the spectra of mycC and LmycC. Furthermore, by considering the intensity of CD maxima, mycC adopts higher ratio of i-motif structures in comparison to LmycC and especially with respect to TmycC. Altogether, CD spectra indicate that introduction of abasic linkers between CCC tracts or substitutions of the first and the fourth CCC tracts with abasic residues are detrimental to i-motif formation. The ambiguity regarding mycC species comprising Watson–Crick base pairs, whose formation is suggested by NMR, but not CD spectra, could be ascribed to different experimental conditions with ten times lower oligonucleotide concentration in the case of CD spectroscopy. Accordingly, the imino signals in ^1^H-NMR spectrum of mycC at 0.2 mM may correspond to intermolecular species, which are disfavored at lower DNA concentrations used in CD spectroscopic analysis.

**Figure 4:**
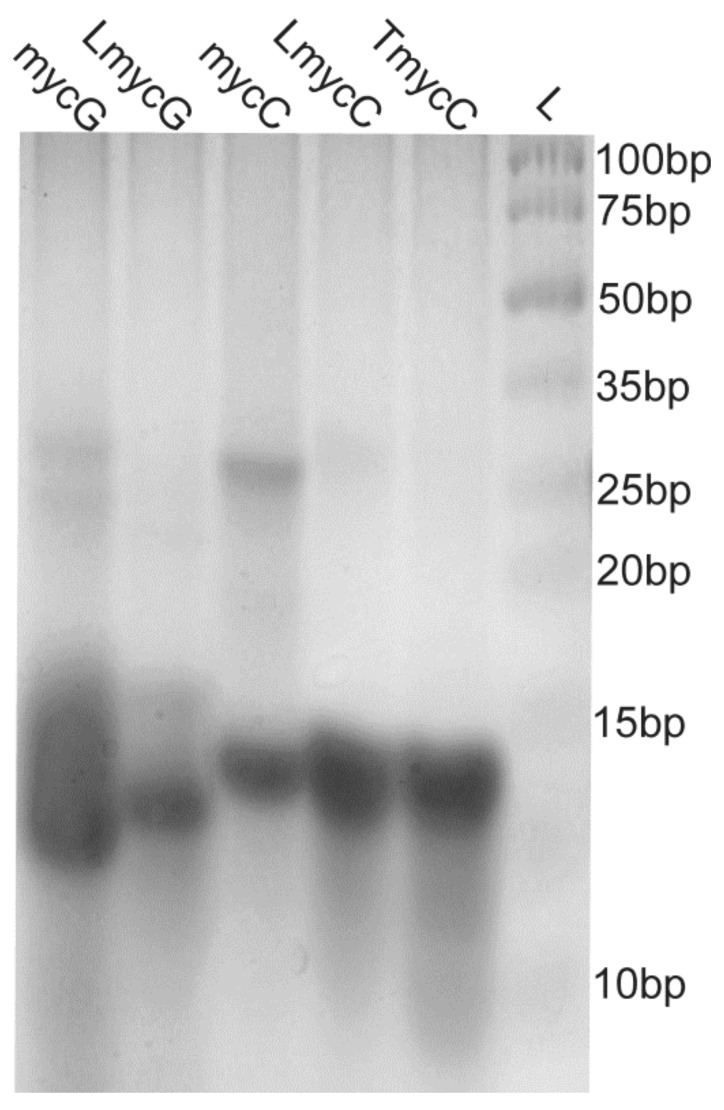
Native polyacrylamide gel electrophoresis (PAGE) of G-rich (mycG and LmycG) and C-rich (mycC, LmycC and TmycC) oligonucleotides at 0.1 mM oligonucleotide concentration per strand. The samples were folded in the presence of 20 mM potassium-phosphate buffer (pH 6.5) and 100 mM KCl and immediately after annealing loaded on gel supplemented with 100 mM KCl. L indicates DNA ladder.

The migration of the studied 27-nt long G- and C-rich DNA oligonucleotides on polyacrylamide gel is similar or slightly faster in comparison to the 15-bp reference double-stranded DNA (Figure 4). This is consistent with the oligonucleotides migrating as unfolded or intramolecular folded species. For mycG much wider band around 15-bp is observed in comparison to the other studied oligonucleotides indicating higher degree of polymorphism. Additional bands for mycG and mycC around 25-bp imply the presence of intermolecular high-order species. Altogether, PAGE results are consistent with mycG and mycC adopting intra- and intermolecular structures, furthermore indicating their higher structural polymorphism in comparison to the modified oligonucleotides.

### 2.2. Individual Oligonucleotide Features Become Apparent Immediately upon Hampering Hybridization between G- and C-rich DNA Strands

**Figure 5:**
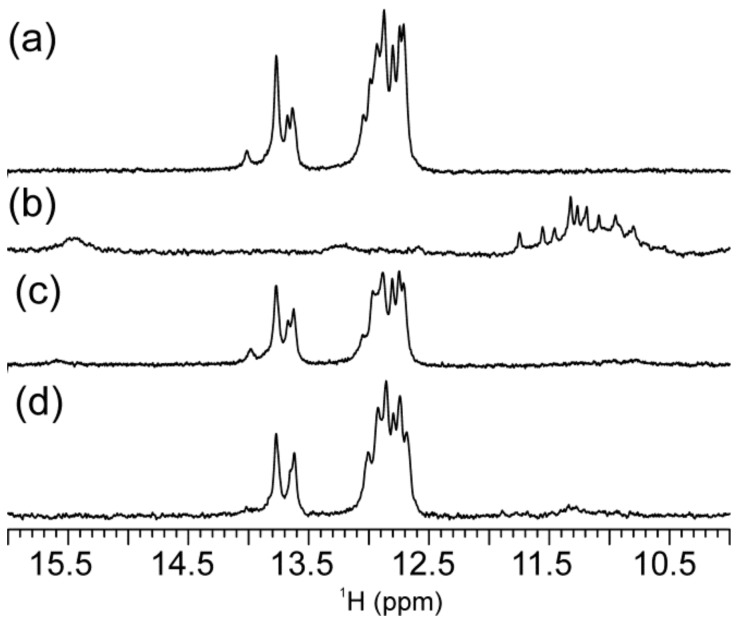
Imino region of ^1^H-NMR spectra of hq recorded on 600 MHz spectrometer at 25 °C, 0.5 mM oligonucleotide concentration per strand and (**a**) 0 mM KCl and pH 6.5, (**b**) 100 mM KCl and pH 5.0, (**c**) 0 mM KCl and pH 5.0 and (**d**) 100 mM KCl and 20 mM potassium-phosphate buffer (pH 6.5).

^1^H-NMR spectrum of hq that corresponds to mycG and mycC at equimolar concentrations exhibits imino proton signals in the range from δ12.60 to 14.00 ppm (Figure 5a). This ^1^H-NMR chemical shift range is characteristic for the Watson–Crick base-pairing. Analysis of the integral values of ^1^H-NMR signals affords the ratio of ca. 3:1 for the imino protons involved in G–C versus A–T base-pairing, which is consistent with formation of double-stranded helix comprising hybridized mycG and mycC. Addition of K^+^ ions into the equimolar mixture of mycG and mycC together with lowering pH is followed by dramatic spectral changes and structural perturbations. Moreover, the ^1^H-NMR signals corresponding to Watson–Crick base-paired imino protons are broadened almost to the baseline (Figure 5b), indicating disintegration of the double-stranded helix. The process of duplex unfolding is accompanied with formation of G-quadruplex and i-motif structures as inferred from the appearance of ^1^H-NMR signals in the range from δ10.82 to 11.76 ppm and at around δ15.5 ppm, which serve as fingerprints of G-quartet and C^+^·C base pair formation, respectively. The spectral features for hq in the presence of K^+^ ion and at lowered pH correspond to the ones observed for individual oligonucleotides mycG and mycC (Figure S2). These results show that at 100 mM KCl and low pH (5.0) hybridization of mycG and mycC at room temperature is diminished in favor of G-quadruplex and i-motif formation. Notably, the lowering of pH alone (Figure 5c) or increase of K^+^ ion concentration at pH 6.5 (Figure 5d) are accompanied with minor spectral changes, suggesting that canonical Watson–Crick base-pairing within the double-stranded helix prevails under those conditions.

**Figure 6:**
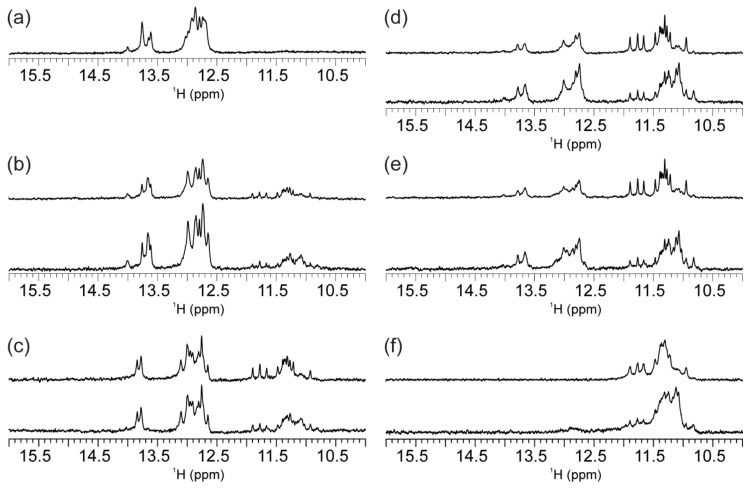
Imino region of ^1^H-NMR spectra of (**a**) hq, (**b**) hqLC, (**c**) hqTC, (**d**) hqLG, (**e**) hqLGLC and (**f**) hqLGTC recorded on 800 MHz spectrometer. In (**b**) (**c**), (**d**), (**e**) and (**f**) upper and lower panels show spectra recorded immediately and one day after annealing, respectively. The spectra were recorded at 25 °C, 0.2 mM oligonucleotide concentration per strand, 20 mM potassium-phosphate buffer (pH 6.5) and 100 mM KCl.

hqLC and hqTC predominantly form double-stranded helix exhibiting imino ^1^H-NMR signals in the same chemical shift range as observed for the parent hq (Figure 6a–c). For both, hqLC and hqTC, additional imino ^1^H-NMR signals are observed in the region characteristic for Hoogsteen-hydrogen bonded guanine residues, consistent with formation of minor G-quadruplex species. Intriguingly, there is a perfect match between the ^1^H-NMR fingerprints of the G-quadruplex species for the hqLC and hqTC with those of mycG. These NMR data indicate that a minor portion of mycG evades hybridization with LmycC or TmycC in hqLC or hqTC, respectively, and adopts G-quadruplex species with the same features in the absence or presence of the C-rich oligonucleotides. Comparison of the ^1^H-NMR spectra of hqLC and hqTC comprising C-rich strand with three and six abasic sites, respectively, shows that more rigorous hindrance in strand complementarity increases amount of G-quadruplexes. As expected, G-quadruplex structures are not formed in the absence of K^+^ ions, as inferred from the ^1^H-NMR spectra of hqTC, where imino signals are observed only in the region characteristic for Watson–Crick base pairs (Figure S3).

hqLG and (the reference) hq differ by G-rich strand, which in the former construct exhibits abasic residues linking the four GGG tracts (Figure 1). ^1^H-NMR spectra of the hqLG comprising LmycG and mycC at 1:1 ratio shows imino ^1^H-NMR signals in the regions characteristic for Watson–Crick base-pairs as well as for Hoogsteen-hydrogen bonded guanines (Figure 6d). This result is consistent with the formation of double-stranded helix and G-quadruplex structures. Similar equilibrium is observed in the case of hqLGLC, where LmycG and LmycC are present in equimolar amounts (Figure 6e). The comparison of imino ^1^H-NMR regions shows only a marginal shift of the equilibrium towards the G-quadruplex species for hqLGLC in comparison to hqLG (cf. Figure 6e,d). In this respect, abasic residues between GGG tracts of LmycG, while not between CCC tracts of mycC are the main cause of diminished hybridization between the G- and C-rich strands in hqLG as well as in hqLGLC. Notably, no G-quadruplex species are observed for hq, which is comprised of mycG and mycC exhibiting exclusively natural residues. Diminished double-stranded helix coupled with appearance of G-quadruplex structures for hqLG and hqLGLC is driven mainly by tendency of LmycG to adopt G-quadruplex structures. The promoted G-quadruplex formation by abasic versus natural residues connecting GGG tracts is demonstrated by comparison of the NMR data on constructs comprising either mycG or LmycG (Figure 6). Moreover, the ratio of G-quadruplex versus double-stranded structures is greater for constructs comprising G-rich strand with abasic loops, regardless of the modifications introduced to the C-rich counterparts. In fact, NMR spectrum of hqLGTC exhibits imino ^1^H signals solely in the range from δ10.81 to 11.90 ppm, consistent with formation of G-quadruplex and absence of double-stranded helix (Figure 6f). The pattern of imino ^1^H-NMR resonances corresponding to G-quadruplex(es) in hqLG, hqLGLC and hqLGTC are same as for LmycG. Hence, the portion of LmycG not involved in hybridization with mycC, LmycC, or TmycC adopts G-quadruplex species with the same structural features as in the absence of the C-rich counterparts.

### 2.3. Presence of Complementary C-Rich Strand Does Not Preclude Transformation of Intra- to Intermolecular G-Quadruplex Species 

**Figure 7:**
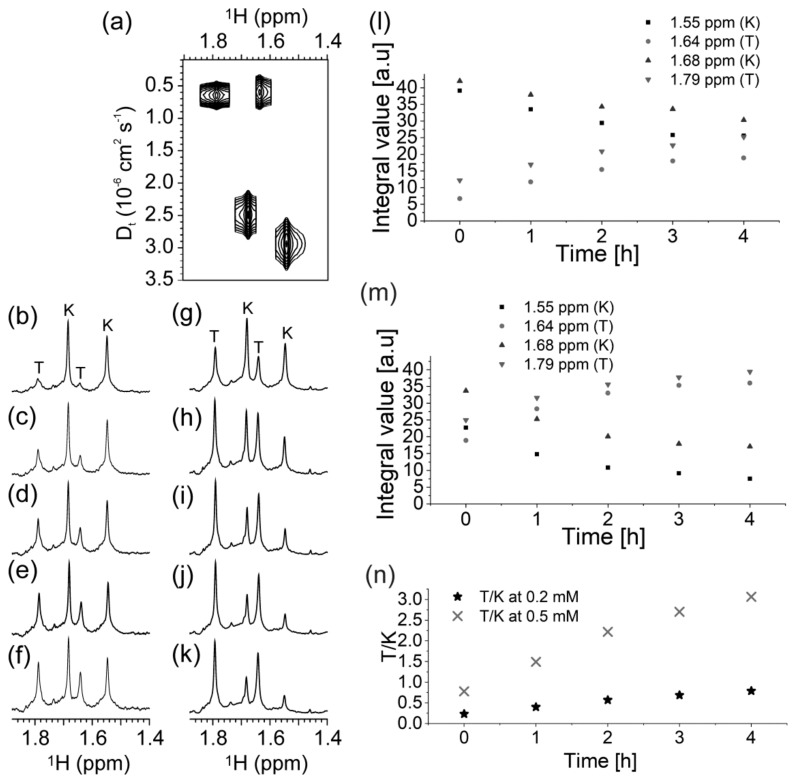
(**a**) Diffusion-ordered spectroscopy (DOSY) NMR spectra of LmycG in the region of methyl group ^1^H-NMR resonances recorded on 800 MHz spectrometer at 25 °C, 100 mM KCl, 20 mM potassium-phosphate buffer (pH 6.5) and 0.5 mM DNA concentration per strand. (**b**–**k**) Time dependence of methyl region of ^1^H-NMR spectra of LmycG recorded on 800 MHz spectrometer at 25 °C, 100 mM KCl, 20 mM potassium-phosphate buffer (pH 6.5). The spectra recorded at 0.2 mM and 0.5 mM oligonucleotide concentration per strand are shown in (**b**–**f**) and (**g**–**k**), respectively. The spectra in (**b**) and (**g**) were obtained immediately after sample preparation, while subsequently the spectra shown in (**c**) and (**h**), (**d**) and (**i**), (**e**) and (**j**), (**f**) and (**k**) were recorded after 1, 2, 3 and 4 h, respectively. The ^1^H signals corresponding to methyl groups of kinetically and thermodynamically favored G-quadruplexes are labelled with ‘K’ and ‘T’, respectively. Time dependence of integral values of ^1^H-NMR signals corresponding to methyl groups of kinetically (‘K’) and thermodynamically (‘T’) favored G-quadruplexes adopted by LmycG at 0.2 mM and 0.5 mM are shown in (l) and (m), respectively. (**n**) The ratio of thermodynamically versus kinetically favored G-quadruplex derived from integrals of ^1^H-NMR signals corresponding to methyl groups.

With the exception of hq, for which G-quadruplex formation is not observed, all other studied constructs are characterized by variation of ^1^H-NMR signals with time (Figure 6). The major changes observed in the 24-h period after annealing relate to the appearance of two sets of imino ^1^H-NMR signals corresponding to two distinct G-quadruplex structures. Notably, the time-dependent structural transformation of mycG and LmycG, that involves the kinetically—versus the thermodynamically—driven formation of G-quadruplex structures occurs in the presence and absence of mycC, LmycC or TmycC. In order to obtain more detailed insights into structural rearrangements of the G-rich strands in the absence and in the presence of a C-rich counterpart, ^1^H-NMR spectra of LmycG and hqLGLC were recorded in 1 hour intervals at two different oligonucleotide concentrations (Supplementary Materials Figures S4 and S5). Imino ^1^H-NMR signals at δ11.66, 11.76, and 11.89 ppm could be assigned to the kinetically predominant G-quadruplex species, while the signal at δ10.81 ppm corresponds to the thermodynamically favored G-quadruplex. The intensity of the ^1^H-NMR signal at δ10.81 ppm with respect to the imino ^1^H-NMR signals of kinetically favored G-quadruplex is higher at 0.5 mM than at 0.2 mM oligonucleotide concentration for both LmycG and hqLGLC. Such DNA concentration-promoted formation of the thermodynamically predominant G-quadruplex species is consistent with intermolecular folding topology. More detailed evaluation of the ratio between different species by means of analyzing imino ^1^H-NMR signals was hindered due to the signal overlap in the range from δ10.90 to 11.50 ppm. Instead, diffusion-ordered spectroscopy (DOSY) NMR spectra were utilized to identify two sets of methyl ^1^H-NMR signals corresponding to the two different G-quadruplexes adopted by LmycG in the absence (Figure 7) and in the presence of C-rich oligonucleotide. Notably, LmycG consists of four GGG tracts linked by single nucleotide residues, which is the same as in N-myc oligonucleotide previously studied in our group [53]. N-myc oligonucleotide adopts monomolecular G-quadruplex with three G-quartets and three single-nucleotide propeller loops at low concentrations of DNA and K^+^ ions. Upon increase in DNA and K^+^ ion concentrations N-myc oligonucleotide forms dimeric G-quadruplex with six consecutively stacked G-quartets. The ^1^H-NMR signals of methyl groups of LmycG with δ1.55 and 1.68 ppm with corresponding apparent diffusion coefficients (Dt) of 2.9 and 2.5 × 10^6^ cm^2^ s^-1^, respectively, are assigned to kinetically predominant monomeric G-quadruplex species. The two additional methyl ^1^H signals of LmycG resonating at δ1.64 and 1.79 ppm with the corresponding apparent Dt of ca. 0.6 × 10^6^ cm^2^ s^-1^ are assigned to thermodynamically favored dimeric G-quadruplex species. The relative ratio between the two species was assessed by analyzing integral values of ^1^H-NMR signals corresponding to methyl groups. The time dependent ratio of the integral values of ^1^H-NMR signals consistently shows faster structural rearrangements at higher oligonucleotide concentration, in line with more intermolecular association.

**Figure 8:**
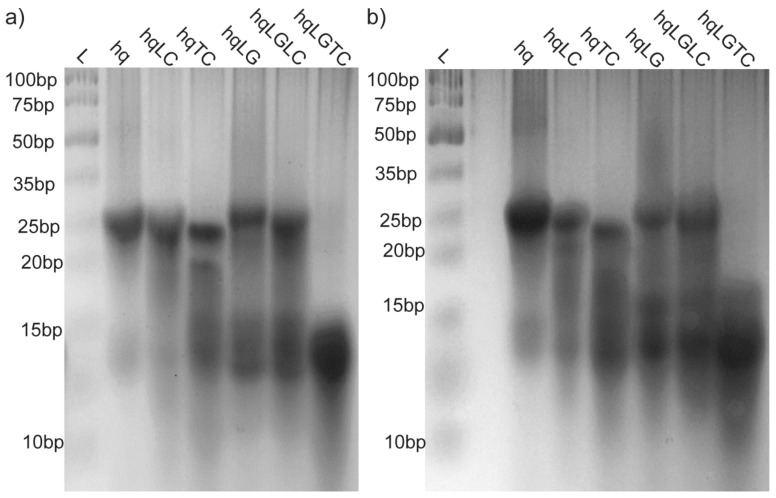
Native PAGE of DNA constructs hq, hqLC, hqTC, hqLG, hqLGLC and hqLGTC at 0.1 mM oligonucleotide concentration per strand (**a**) immediately and (**b**) 2 days after annealing procedure. The samples were folded in the presence of 20 mM potassium-phosphate buffer (pH 6.5) and 100 mM KCl and loaded on gel supplemented with 100 mM KCl. L indicates DNA ladder.

For all constructs except the hqLGTC band around 25-bp is observed on polyacrylamide gel corresponding to double-stranded structures (Figure 8). Additional species are resolved on the gel, including the ones migrating at 15-bp or lower corresponding to unfolded and intramolecular folded species. The smearing in the region above 15-bp and below 25-bp is more intense for 2 days old samples and thus consistent with a gradual transformation of intramolecular to larger intermolecular species. Smearing above 25-bp is observed for hq and hqLG indicating that portion of mycC that evades hybridization with G-rich strand might form high-order species. On the other hand, the lack of bands above 25-bp in the case of all constructs, other than hq and hqLG, implies that intermolecular interactions result in the formation of dimeric species utmost.

To obtain further structural insights melting profiles of the studied oligonucleotides and constructs were monitored by UV absorbance at 295 nm and 260 nm (Figures S6 and S7, Table S1). The difference between heating and cooling profiles is more prominent for constructs than for individual oligonucleotides, but is nevertheless observed for all. For mycG, two transitions in melting profile are evident, i.e., at ca. 34 and 84 °C, while the high thermal stability of LmycG precluded determination of T_m_ up to 95 °C. The melting profiles monitored by absorbance at 260 nm are consistent with mycC and TmycC adopting the most and the least stable high-order structures amongst the C-rich oligonucleotides. Transition in the range from 83 to 85 °C reflecting melting of mycG G-quadruplex structure(s) is observed for hq, hqLC and hqTC. Additionally, apparent T_m_ of duplex structures in case of hq, hqLC and hqTC estimated from the melting profiles monitored by absorbance at 260 nm are ca. 71, 50, and 32 °C, respectively. For the constructs comprising LmycG as the G-rich strand T_m_ of G-quadruplex structure(s) could not be assigned. The apparent T_m_ of duplex structures in case of hqLG, hqLGLC and hqLGTC are ca. 42, 43, and 14 °C, respectively, however these values should be considered with precaution as they expectedly correspond to the average of several folding/unfolding processes.

## 3. Discussion

G- and C-rich DNA can adopt non-canonical structures, which are considered as key (regulatory) elements in biological processes and are furthermore relevant for development of new therapeutic approaches relying on targeting of four-stranded DNA structures [55]. Biophysical parameters such as temperature, pH, presence of cations and cosolutes as well as biological processes such as DNA transcription have been studied to demonstrate their effect on the equilibrium between double-stranded and four-stranded DNA structures [29,30,31,51,56,57,58]. First of all, however, the tendency of a G- and C-rich DNA to form G-quadruplex and i-motif structures, respectively, depends on the nucleotide sequence. By varying length and composition of the segments (sequence) between G- and C-tracts it has been shown that longer loops favor double-stranded helix and diminish G-quadruplex formation [26]. Furthermore, it was shown that duplex formation was coupled with unfolding of a single G-quadruplex in the case of only one thymine residue between GGG tracts. On the other hand, two G-quadruplexes conformers were identified when several thymine residues were introduced between GGG tracts. Herein we used natural-to-abasic residue modifications to focus on the interplay of G- and C-rich DNA strands’ hybridization versus non-canonical structure formation by individual strands. The reference 27-bp construct named hq comprises central 17-bp segment originating from the first intron of *N-myc* gene. Moreover, hq corresponds to equimolar mixture of complementary G- and C-rich oligonucleotides i.e., mycG and mycC, respectively. Five constructs were derived from hq by using variants of mycG and mycC carrying substitutions of natural with abasic residues between GGG tracts, CCC tracts and instead of the first and the terminal CCC tracts (Figure 1). Detailed NMR spectral analysis showed that introduction of abasic residues results in equilibrium in which G-rich strand evades hybridization with C-rich strand and subsequently folds into a G-quadruplex structure(s). We focused on constructs comprising 17-bp long G/C-rich central segment, which is related to particular biologically relevant region, i.e. the first intron of *N-myc* gene, while it may also be considered as a model for elucidating general features of non-canonical structure in context of double-stranded helix. Moreover, the studied constructs comprised G-quadruplex/i-motif-forming region extended at both, 5′- and 3′-ends with five base-pair, which roughly corresponds to half of the length required for B-form helix to make the whole turn. Interestingly, while we observed G-quadruplex formation in all constructs carrying modifications, there was an absence of species exhibiting G-quadruplex and/or i-motif structures ‘framed’ within Watson–Crick base-paired termini of G- and C-rich strands, which demonstrates that formation of the non-canonical structures precludes hybridization of complementary regions. Considering that the studied constructs comprise only five base-pairs at both, 5′- and 3′-ends with respect to the central G/C-rich segment, longer constructs would be required to further explore G-quadruplex and i-motif features in context of double-stranded DNA.

^1^H-NMR spectra of hqLG reveal the formation of double-stranded helix and G-quadruplex structures, whereas only the former are observed in the case of hq. Considering that hqLG is distinguished from hq by abasic instead of natural residues between the four GGG tracts the diminished hybridization of G- and C-rich strand is caused by the abasic linkers between GGG tracts. hqLC and hqLGLC both comprise LmycC exhibiting abasic residues between CCC tracts, whereas hqLGLC contains abasic linkers also between GGG tracts. The observation of higher G-quadruplex species ratio for hqLGLC in comparison to hqLC demonstrates that abasic residues in G-rich strand hinder formation of double-stranded helix by not only diminishing complementarity to C-rich strand but also increasing G-quadruplex-forming potential of the G-rich strand. Notably, the abasic residues between GGG tracts in LmycG were at the outset expected to contribute to flexibility of loops in terms of G-quadruplex structure formation. Moreover, in oppose to natural residues, abasic linkers between GGG tracts exert fewer interactions in terms of stacking and H-bonding, while in parallel minimize steric effects that may hinder G-quadruplex formation. The effect of abasic residues in LmycG in promoting G-quadruplex formation in correspondence to mycG is consistent with ^1^H-NMR and CD spectral results. Moreover, the ratio of G-quadruplex versus double-stranded structures is greater for constructs comprising G-rich strand with abasic in comparison to natural residues, regardless of the modifications introduced to the C-rich counterparts. In fact, only G-quadruplex structures are observed for hqLGTC, whereas for hqTC there is equilibrium between G-quadruplex and double-stranded helix.

The mutual exclusivity of G-quadruplex and i-motif formation within complementary strands was demonstrated by mechanical unfolding profiles of four-stranded structures in variety of double-stranded DNA templates [59]. Moreover, by demonstrating a negative correlation between the simultaneous formation of four-stranded structures in complementary strands and a decrease in offset between G-quadruplex- and i-motif-forming regions, steric hindrance was established as the cause for mutual exclusivity. CD spectroscopic data on the series of C-rich oligonucleotides mycC, LmycC, and TmycC show that their propensity to form i-motif structure(s) decreases upon abasic substitutions. Moreover, abasic linkers between CCC tracts decrease i-motif formation, but to a lesser extent than cytosine-to-abasic substitutions in the first and the terminal CCC tracts. While for the oligonucleotides series the effects of modifications in C-rich strands were directly observable, they were less resolved in context of constructs. Moreover, ^1^H-NMR data are consistent with only marginally higher ratio of G-quadruplex versus double-stranded structures for hqLGLC in comparison to hqLG, while in case of hqTC only G-quadruplex structure(s) are observed. In this respect, the highest G-quadruplex ratio for hqTC correlates to the lowest i-motif-forming potential of TmycC in comparison to mycC and LmycC. On the other hand, LmycC is less prone to adopt i-motif structure(s) in comparison to mycC and yet ratio of G-quadruplex versus duplex structures is only marginally higher for hqLGLC than hqLG. Thus, the diminished duplex formation and higher ratio of G-quadruplex in constructs comprising C-rich strand(s) with abasic modifications (Lmyc and TmycC) is mainly caused by the impairment of complementarity to G-rich strand(s) rather than the differences in i-motif-forming potential.

The time-dependent changes of ^1^H-NMR signals for mycG, LmycG and for all but hq constructs demonstrate that the investigated G-rich oligonucleotides undergo structural transitions. Moreover, the transformation of initially formed intramolecular G-quadruplex into the thermodynamically favored intermolecular G-quadruplex occurs even in the presence of C–rich counterpart. Thus, transformation of intra- to intermolecular G-quadruplex predominates over unfolding of intramolecular G-quadruplex coupled with hybridization of ‘free’ G- and C-rich strands. In this respect, propensity of Watson–Crick base-pairing between G- and C-rich strands is insufficient to preclude conversion between the structural forms comprising G-quartets. This phenomenon might be characteristic of DNA disposed to form intermolecular G-quadruplexes such as mycG and LmycG, especially when considering prominent nucleotide sequence-dependence of G-rich DNA structures [60,61,62]. Interestingly, previous study showed that G-rich c-myc sequence detaches from its complementary strand due to its tendency for G-quadruplex formation in a process coupled with re-hybridization of C-rich strand with a new (labelled) G-rich c-myc strand [35]. In light of our results and without considering the differences in nucleotide sequences used previously and herein, it is tempting to speculate whether or how apparently minor differences between unlabeled/non-modified and labelled/modified G-rich strands influence their G-quadruplex forming potential and furthermore hybridization with C-rich strand. We show that the abasic linkages introduced between GGG tracts boost G-quadruplex formation enabling G-rich strand to (in part) evade hybridization to C-rich strand. Other modifications of G-rich strand might diminish folding of non-canonical structures thereby promoting hybridization to C-rich strand. These conceptions tend to incite consideration when designing G- to C-rich DNA ‘perfect-match’ solely on Watson–Crick complementarity.

The G-rich region of mycG consists of four GGG tracts linked by single nucleotide residues and matches the one in N-myc oligonucleotide previously studied in our group [53]. At low DNA and K^+^ ions concentration N-myc oligonucleotide adopts monomolecular G-quadruplex with three G-quartets, while upon increase in DNA and K^+^ ion concentrations dimeric G-quadruplex with six consecutively stacked G-quartets is formed. Notably, the dimeric N-myc G-quadruplex does not form by stacking interactions between outer G-quartets of monomeric units, but is a result of intertwinement of two DNA molecules, which requires annealing of the sample. Hence, the time dependent structural changes of mycG and LmycG cannot be trivially explained. However, there have been reports on gradual time-dependent formation of dimeric G-quadruplex with intertwined strands from initially present monomeric G-quadruplex species [63]. Accordingly, formation of intertwined dimeric G-quadruplex by mycG and LmycG may proceed without requirement of temperature treatment. From this point of view the longer 5′- and 3′-ends of mycG and LmycG in comparison to N-myc oligonucleotide seem to promote the formation of dimeric G-quadruplexes. Notably, an alternative manner of dimeric G-quadruplex formation with respect to intertwinement of two mycG or LmycG would be association of monomeric G-quadruplex units through outer-G-quartet-stacking. However, this process seems unlikely, as 5′- and 3′-end overhanging residues are expected to sterically hinder stacking interactions at outer G-quartets’ interfaces [64,65]. Additionally, no band above 25-bp is observed by PAGE analysis for all constructs except hq and hqLG. These results demonstrate absence of association of monomeric G-quadruplex units via G-quartet stacking, which further supports intertwinement as mechanism of dimerization of G-rich strands. The transformation of monomeric to dimeric G-quadruplex corroborates the importance of interactions between G-rich segments of different DNA strands in the fundamental processes such as DNA recombination [66]. In addition, hybrid DNA-RNA G-quadruplex structures are involved in DNA replication [67], transcription [68] and protection of telomere ends [69]. The present study provides insights that may enable better understanding of DNA-DNA dimeric G-quadruplexes in correspondence to DNA-RNA hybrid G-quadruplexes.

## 4. Materials and Methods

### 4.1. Oligonucleotides

DNA oligonucleotides were synthesized on an Expedite 8909 synthesizer (Applied Biosystems, Foster City, CA, USA) with the use of standard phosphoramidite chemistry and deprotected with the use of aqueous ammonia. RP-HPLC was used for purification, while gel-filtration through a Sephadex G25 column was used for desalting of the samples. The pH was regulated by addition of HCl and LiOH solutions and subsequent addition of buffer solution. If not stated otherwise, the samples in the presence of K^+^ ions were prepared at the final concentrations of KCl and potassium-phosphate buffer (pH 6.5) at 100 mM and 20 mM, respectively. The annealing of the samples before spectroscopic and PAGE analysis included 5 min incubation at 90 °C immediately followed by cooling in an ice-water bath for 20 min.

### 4.2. NMR Spectroscopy

NMR data were collected on Varian NMR Systems spectrometers (Varian Inc., Palo Alto, CA, USA) operating at 600 MHz and 800 MHz. ^1^H, NOESY and DOSY spectra in 90%/10% H_2_O/^2^H_2_O were acquired at 25 °C with the use of DPFGSE solvent suppression method. Fifteen different gradient strengths (2.69–67.28 G cm^−1^) were used in diffusion experiments. Mixing times used in NOESY experiments were between 80 and 200 ms. NMR spectra were processed and analyzed with the use of VNMRJ (Varian Inc., Palo Alto, CA, USA) software. DNA oligonucleotide concentrations in NMR samples were between 0.2 and 0.5 mM per strand. 

### 4.3. CD Spectroscopy

CD experiments were carried out on Applied Photophysics Chirascan CD (Surrey, UK) spectrometer at room temperature over the 200–340 nm wavelength range with the use of 0.1 cm path-length quartz cells. CD samples were at 0.02 mM in DNA oligonucleotide concentration per strand and the concentrations of KCl and potassium-phosphate buffer (pH 6.5) were 100 mM and 20 mM, respectively.

### 4.4. Native PAGE

The samples together with a GeneRuler Ultra Low Range DNA ladder with 10–300 base pairs (Fermentas, Waltham, MA, USA) were loaded and resolved on 20% native PAGE gels, which were supplemented with 100 mM KCl. Stains All (Sigma-Aldrich, Rockville, MD, USA) was used for staining of the gels after performing electrophoresis. The samples loaded on the gels were at 0.1 mM in oligonucleotide concentration per strand, 100 mM in KCl and 20 mM in potassium-phosphate buffer (pH 6.5).

### 4.5 UV Spectroscopy

Melting experiments were performed on Cary 3500 UV-Vis spectrophotometer (Agilent Technologies, Santa Clara, CA, USA). A temperature range from 5 to 95 °C was scanned while monitoring absorbances at 260 nm and 295 nm with 0.5 °C min^−1^ melting/annealing rate. Sample solutions contained 100 mM KCl and 20 mM K-phosphate buffer (pH 6.5). Measurements were made in 1 cm path-length cells.

## 5. Conclusions

This study shows how the polymorphic nature of the G-rich DNA oligonucleotides can manifest in the formation of two G-quadruplex species with distinct folding kinetics and molecularities in the absence and, more importantly, the presence of a complementary C-rich DNA strand. Such G-rich strand associations cannot be generalized as they are expectedly tightly related to DNA sequence context. Nevertheless, the demonstrated prevalence of ‘oligonucleotide-alone’ features in the equimolar mixtures of complementary strands shows that interactions between G-quartets’ comprising structures is an effective means for the G-rich strand to evade intermolecular Watson–Crick base-pairing.

## Supplementary Materials

The following are available online, Figure S1: ^1^H-NMR spectra of mycG, Figure S2: 1H-NMR spectra of hq, mycG and mycC, Figure S3: 1H-NMR spectra of hqTC, Figure S4: Time-dependence of ^1^H-NMR signals for LmycG at different concentrations, Figure S5: Time-dependence of 1H-NMR signals for hqLGLC at different concentrations, Figure S6: UV melting profiles of oligonucleotides, Figure S7: UV melting profiles of constructs, Table S1: Melting transitions for the studied oligonucleotides and constructs.
