# Supplementary material for: Chasing Particularities of Guanine- and Cytosine-Rich DNA Strands
Source: Molecules. 2020 Jan 21;25(3):434. doi: 10.3390/molecules25030434 (PMC7037129; doi:10.3390/molecules25030434)
Supplement: Supplementary file 1 [file molecules-25-00434-s001.pdf]

Supplementary Data

Chasing particularities of guanine- and cytosine-rich DNA strands

Marko Trajkovski [1,*] and Janez Plavec [1,2,3]

[1] Slovenian NMR Centre, National Institute of Chemistry, Hajdrihova 19, Ljubljana, SI-1000, Slovenia

[2] EN-FIST Centre of Excellence, Trg OF 13, SI-1000 Ljubljana, Slovenia

[3] Faculty of Chemistry and Chemical Technology, University of Ljubljana, Večna pot 113, p. p. 537, SI-1000 Ljubljana, Slovenia

* To whom correspondence should be addressed: marko.trajkovski@ki.si; Tel: +386 1 47 60 505;

Present Address: Marko Trajkovski, Slovenian NMR Centre, National Institute of Chemistry, Hajdrihova 19, Ljubljana, SI-1000, Slovenia

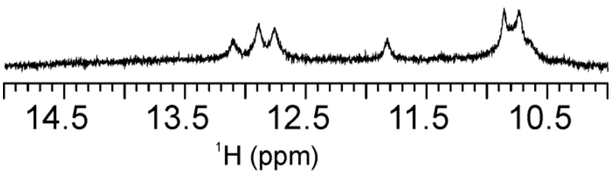

Fig. S1. Imino region of ^{1}H NMR spectra of mycG recorded on 800 MHz spectrometer at 25 °C, 0.2 mM oligonucleotide concentration per strand and pH 6.5.

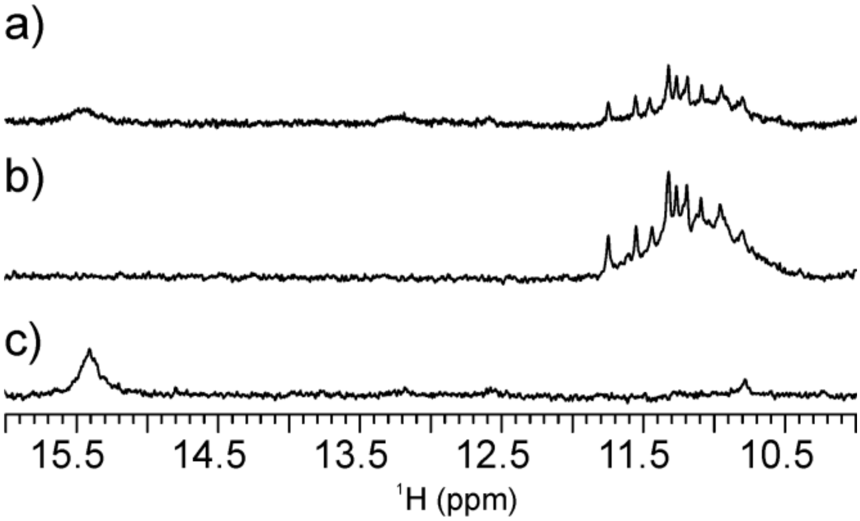

Fig. S2. Imino region of ^{1}H NMR spectra of a) hq, b) mycG and c) mycC recorded on 600 MHz spectrometer at 25 °C, 0.2 mM oligonucleotide concentration per strand, 100 mM KCl and pH 5.0.

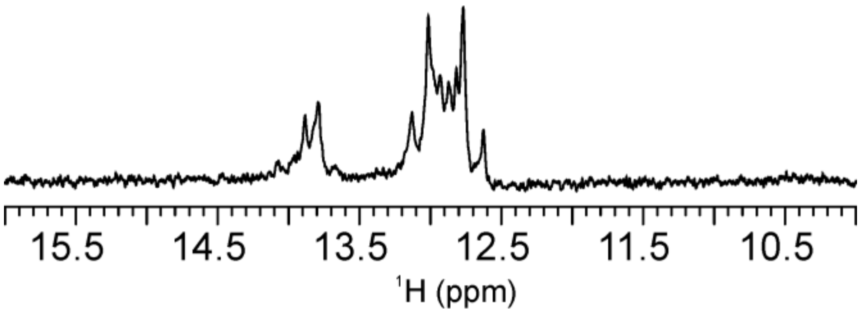

Fig. S3. Imino region of ^{1}H NMR spectra of hqTC construct recorded on 800 MHz spectrometer at 25 °C, 0.2 mM oligonucleotide concentration per strand, 20 mM PIPES buffer (pH 6.8) and 100 mM LiCl.

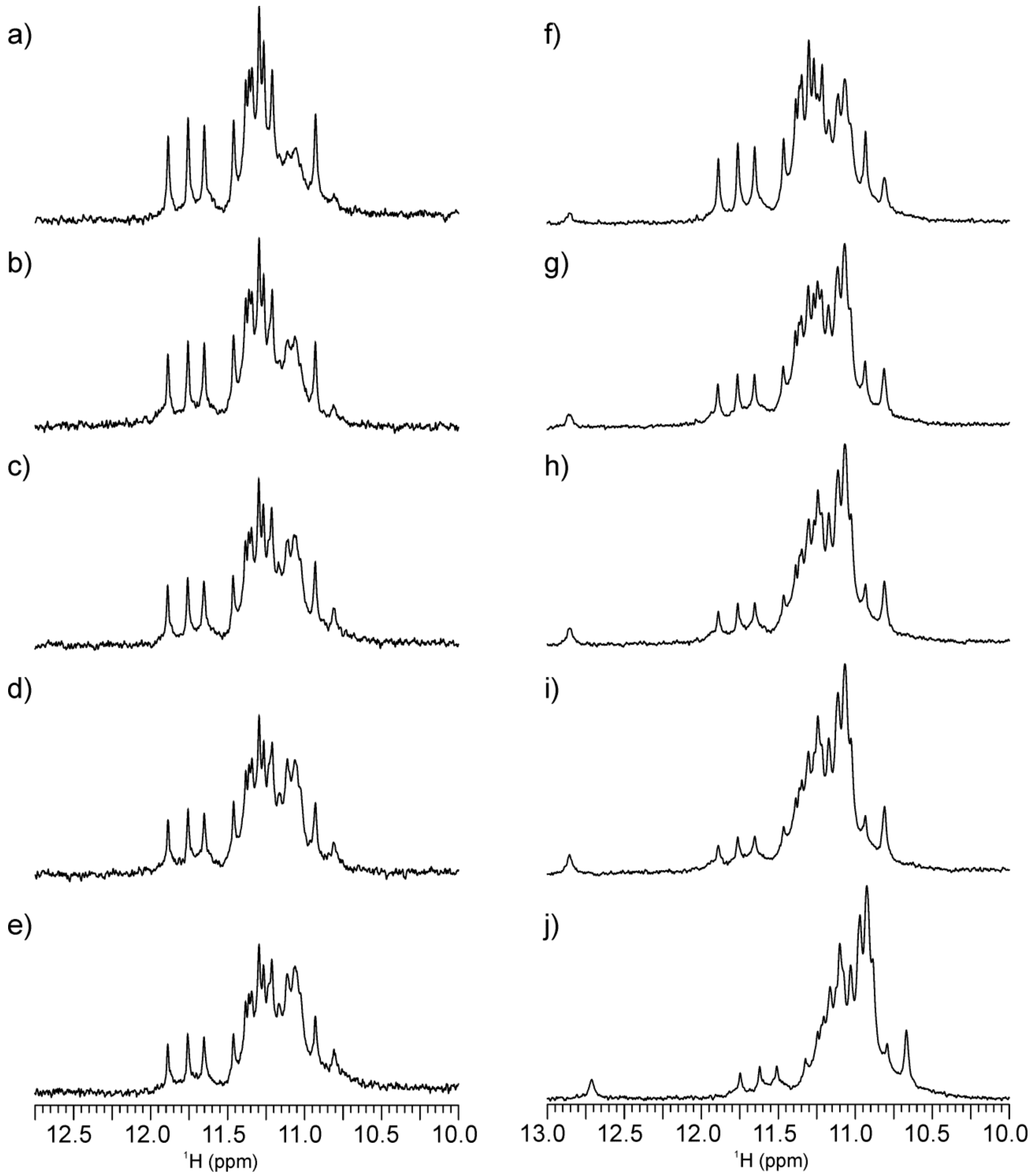

Fig. S4. Time dependence of imino region of ^{1}H NMR spectra of LmycG at a-e) 0.2 mM and at f-j) 0.5 mM oligonucleotide concentration per strand. The spectra shown in a) and f) were obtained immediately after heat-and-anneal procedure, while the spectra shown in b) and g), c) and h), d) and i), e) and j) were recorded 1, 2, 3 and 4 hours after the annealing, respectively. The spectra were recorded on 800 MHz spectrometer at 25 °C, 100 mM KCl and 20 mM potassium-phosphate buffer (pH 6.5).

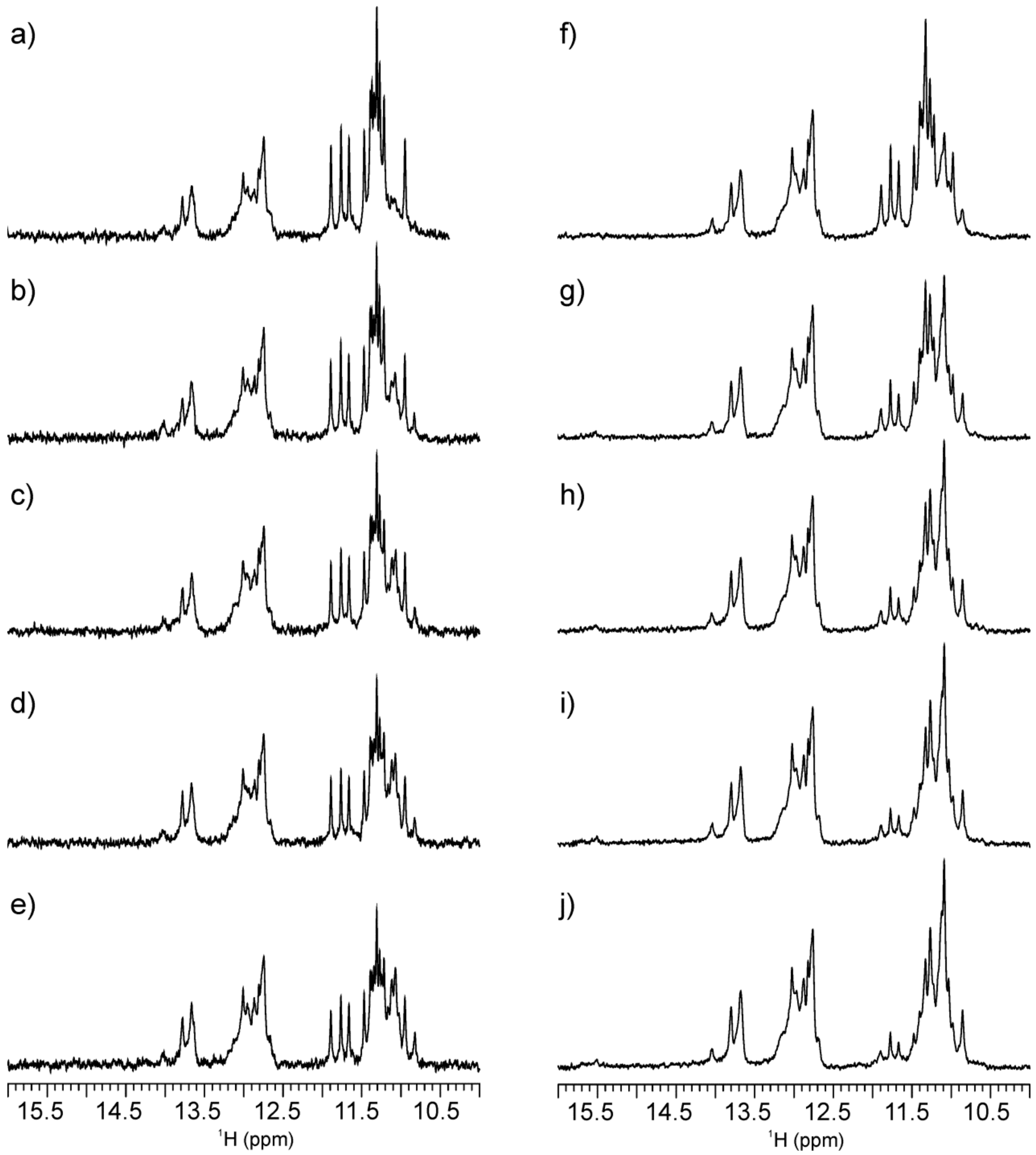

Fig. S5. Time dependence of imino region of ^{1}H NMR spectra of hqLGLC construct at a-e) 0.2 mM or f-j) 0.5 mM oligonucleotide concentration per strand. The spectra shown in a) and f) were obtained immediately after heat-and-anneal procedure, while the spectra shown in b) and g), c) and h), d) and i), e) and j) were recorded 1, 2, 3 and 4 hours after annealing, respectively. The spectra were recorded on 800 MHz spectrometer at 25 °C, 100 mM KCl and 20 mM potassium-phosphate buffer (pH 6.5).

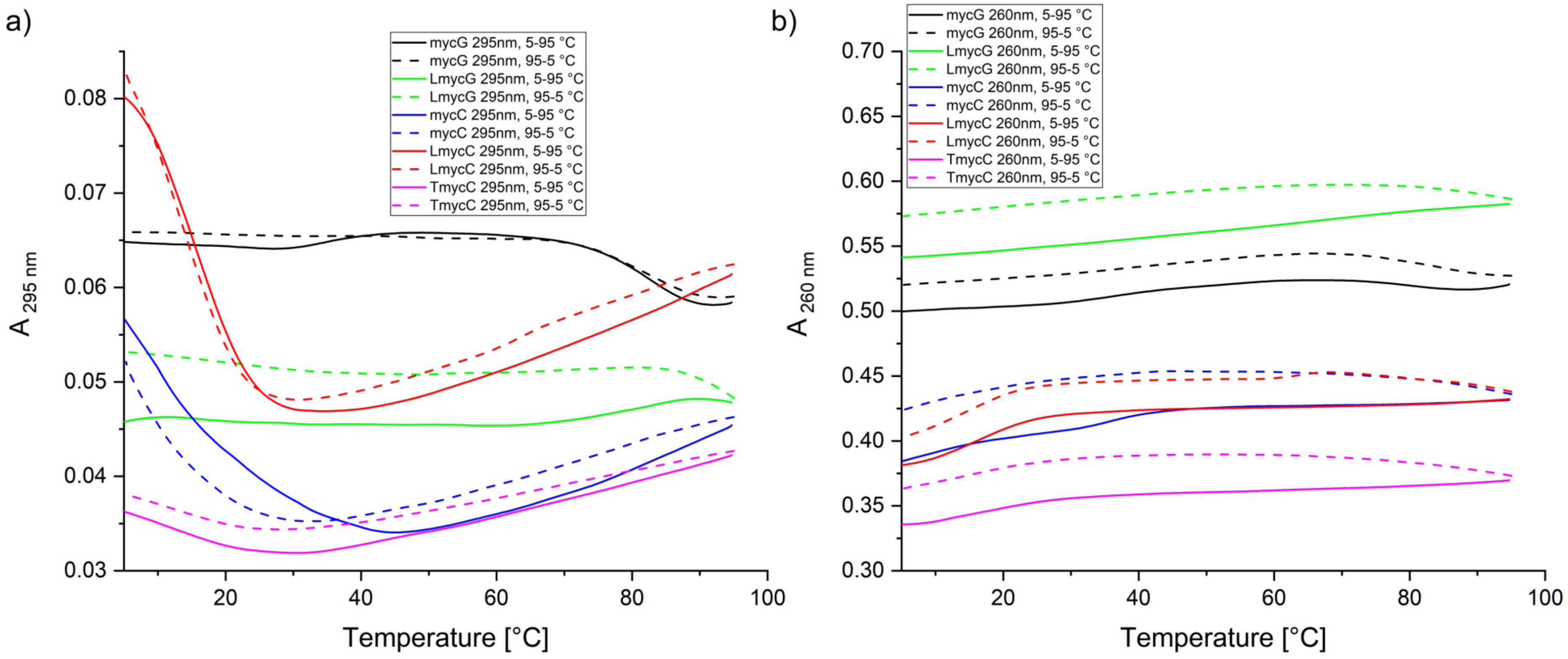

Fig. S6. UV melting profiles of the studied oligonucleotides at 2 µM oligonucleotide concentration per strand, 100 mM KCl and 20 mM K-phosphate buffer (pH 6.5) at a) 295 nm and b) 260 nm.

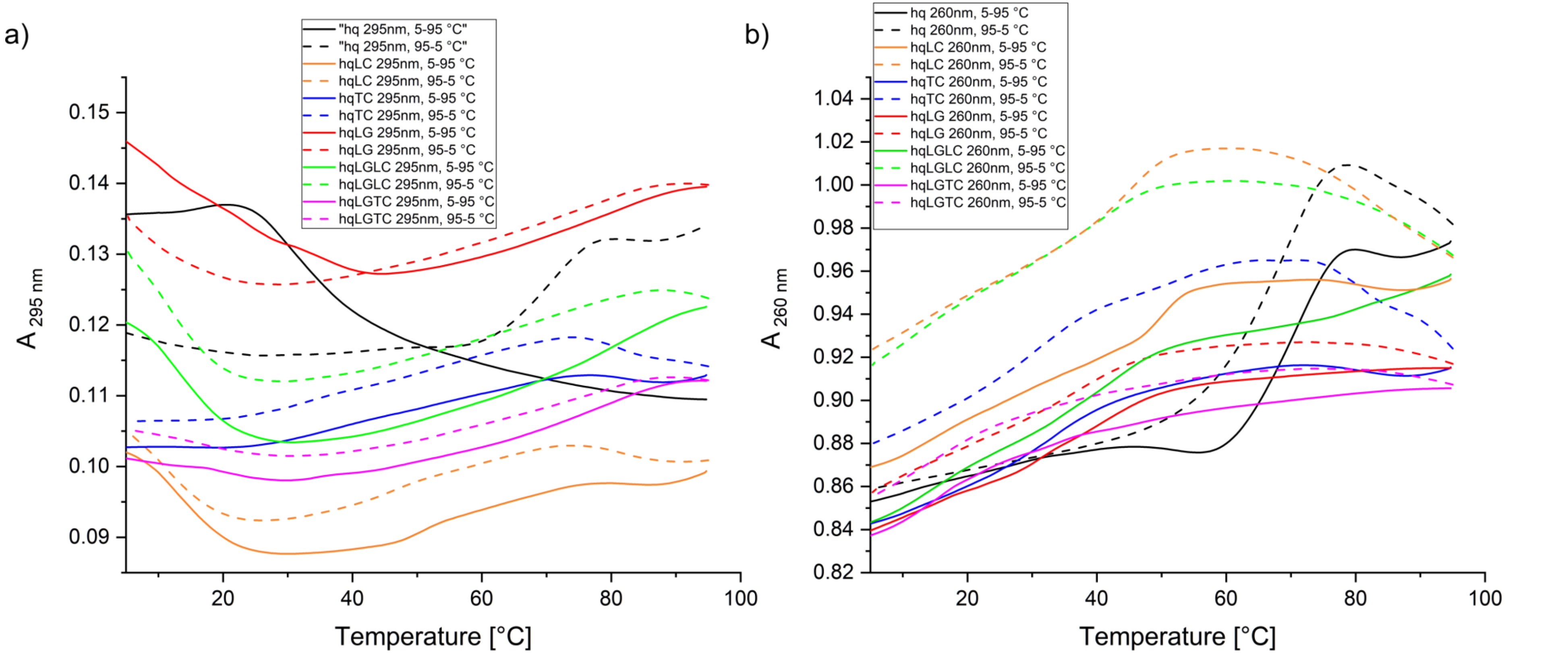

Fig. S7. UV melting profiles of the studied constructs at 2 µM oligonucleotide concentration per strand, 100 mM KCl and 20 mM K-phosphate buffer (pH 6.5) at a) 295 nm and b) 260 nm.

Table S1. Apparent temperatures of melting transitions for the oligonucleotides alone and in constructs assessed from the first derivative of the absorbance signal at 295 and 260 nm.*

<table>
<tr><td></td><td colspan="4">heating (5-95 °C)</td><td colspan="4">cooling (95-5 °C)</td></tr>
<tr><td></td><td colspan="8">Transitions from monitoring absorbance at 295nm</td></tr>
<tr><td>mycG</td><td colspan="3">34 °C</td><td>84 °C</td><td colspan="4">84 °C</td></tr>
<tr><td>LmycG</td><td colspan="4">n.d.</td><td colspan="4">n.d.</td></tr>
<tr><td>mycC</td><td colspan="4">n.d.</td><td colspan="4">n.d.</td></tr>
<tr><td>LmycC</td><td colspan="4">16 °C</td><td colspan="4">13 °C</td></tr>
<tr><td>TmycC</td><td colspan="4">10 °C</td><td colspan="4">11 °C</td></tr>
<tr><td>hq</td><td colspan="2">72 °C</td><td colspan="2">84 °C</td><td colspan="2">70 °C</td><td colspan="2">83 °C</td></tr>
<tr><td>hqLC</td><td>13 °C</td><td colspan="2">51 °C</td><td>85 °C</td><td>11 °C</td><td colspan="2">46 °C</td><td>82 °C</td></tr>
<tr><td>hqTC</td><td colspan="3">12 °C</td><td>83 °C</td><td colspan="3">12 °C</td><td>81 °C</td></tr>
<tr><td>hqLG</td><td colspan="4">n.d.</td><td colspan="4">n.d.</td></tr>
<tr><td>hqLGLC</td><td colspan="4">13 °C</td><td colspan="4">12 °C</td></tr>
<tr><td>hqLGTC</td><td colspan="4">14 °C</td><td colspan="4">15 °C</td></tr>
<tr><td colspan="9"></td></tr>
<tr><td></td><td colspan="4">heating (5-95 °C)</td><td colspan="4">cooling (95-5 °C)</td></tr>
<tr><td></td><td colspan="8">Transitions from monitoring absorbance at 260 nm</td></tr>
<tr><td>mycG</td><td colspan="3">36 °C</td><td>82 °C</td><td colspan="4">85 °C</td></tr>
<tr><td>LmycG</td><td colspan="4">n.d.</td><td colspan="4">n.d.</td></tr>
<tr><td>mycC</td><td colspan="4">36 °C</td><td colspan="4">n.d.</td></tr>
<tr><td>LmycC</td><td colspan="4">16 °C</td><td colspan="3">14 °C</td><td>65 °C</td></tr>
<tr><td>TmycC</td><td colspan="2">12 °C</td><td colspan="2">20 °C</td><td colspan="4">14 °C</td></tr>
<tr><td>hq</td><td colspan="2">71 °C</td><td colspan="2">83 °C</td><td colspan="2">70 °C</td><td colspan="2">83 °C</td></tr>
<tr><td>hqLC</td><td colspan="3">50 °C</td><td>81 °C</td><td>46 °C</td><td colspan="2">61 °C</td><td>83 °C</td></tr>
<tr><td>hqTC</td><td colspan="3">33 °C</td><td>81 °C</td><td colspan="3">33 °C</td><td>81 °C</td></tr>
<tr><td>hqLG</td><td colspan="4">42 °C</td><td colspan="4">37 °C</td></tr>
<tr><td>hqLGLC</td><td colspan="2">14 °C</td><td colspan="2">43 °C</td><td colspan="2">11 °C</td><td colspan="2">42 °C</td></tr>
<tr><td>hqLGTC</td><td colspan="4">16 °C</td><td colspan="4">14 °C</td></tr>
</table>

* Considering the complex equilibria of different (intermolecular) structures and the error related to using first derivative of the absorbance signal for melting analysis the apparent temperatures of melting transitions might not correspond to T_m.
